# Indents

**Published:** 1902-01-15

**Authors:** 


### INDENTS.

**☞ Brief Articles of Technical or Scientific Value will receive notice and comment. Contributions invited.**

Painless Removal of Tooth-Enamel.—Removing the enamel should not cause any pain worth mentioning. By placing a short piece of rubber tubing which fits tightly around the tooth, and leaving on over night so that the gum may be pressed back, the enamel may be removed qutte painlessly without even causing the gums to bleed.—*Dominion Dental Journal.*

Pulp Mummification.—Pulps being in warm, moist mouths, there is no remedy known to chemistry that will, under such circumstances, make a mummy that will remain a mummy. Put a mummy under the pyramids of Egypt, and in that dry atmosphere it will remain a mummy, but the pulp of a tooth in the mouth does not act that way.—Dr. Bogue, in *Items of Interest.*

A New Alloy.—According to the *Zahntechnische Reform*, one hundred parts of copper and six of antimony form an alloy with properties very much like those possessed by gold. The antimony is added to the melted copper and the whole is covered with charcoal ashes, magnesia and lime. It can be rolled and worked like gold; its color is like that of gold, and it is said not to become dark with age.

Separating the Teeth.—When a great deal of separation is needed, partially excavate the cavity and pack very firmly with cotton saturated with chloro-percha. It may remain two or three weeks if needed. The teeth will be well separated, the cavity easy of access and perfectly protected for the time being by the chloro-percha, while the cotton can not become foul.—C. O. Hood, in *Dominion Dental Journal.*

Oil of Cinnamon and Thymol as Antiseptic.—F. A. Howorth, in the *Pharmaceutical Journal*, recommends the following combination for obtunding sensitive dentin, and also as an antiseptic root canal dressing. Take of thymol one dram, and dissolve it in an equal quantity of alcohol or chloroform, and then add one dram of the oil of cinnamon. He also suggests that equal parts of menthol and thymol, rubbed together until they form a liquid, make a good antiseptic dressing.

Articulation and Occlusion.—The degree of usefulness and longevity of the artificial substitute depends greatly upon such formation of the articulating surfaces, and in the posterior region the arrangement of cusps and sulci in their relation to the antagonizing teeth, as will restore their normal functions. The arrangement should provide for correct position not only when the teeth are in direct occlusion, but also in their articulation or the act of bringing them into occlusion.—Goslee, in *Items of Interest.*

Dryess of Cavity Essential in Applying Arsenic.—If arsenic is applied to a cavity which has not been thoroughly dried and freed of decayed dentin and food *debris*, the immediate effect will be exceedingly painful. Arsenic can not act if the avenues of penetration are obstructed with disorganized matter. In order to obtain a complete result from an application of arsenous oxid, the cavity in which it is placed should be perfectly dry and clean, otherwise it will simply act as an irritant and will cause much suffering.

The Use of Drills in Opening Cavities.—Dr. Osmun in the December *Items*, calls attention to the fact that drills used in the engine for opening into carious teeth, produce so much vibration as to inflict needless pain, or at least to exite grave apprehension of impending pain. He suggests the use of small disks or stones kept wet with a stream of warm water. Avoid running the engine at too high speed and have the water running freely all the time, This will make the operation less painful and also secure good smooth margins.

Chinosol (potassium oxy-quinoline sulphonate) is a yellowish powder, antiseptic and powerfully deodorant. It is not caustic, coagulant or injurious to instruments. Its solutions have a strong astringent taste with an aromatic odor, and are not poisonous. It is also a good styptic. Tablets containing eight grains are marketed, one of which dissolved in a pint of distilled water makes a solution of 1 to 1200, which is sufficiently strong for use as a mouth wash or gargle. Stronger solutions are necessary for lotions or circumscribed local application. —F. A. Howorth, in *Phar. Journal.*

To Secure Proper Color for Inlays.—The most beautiful work I have ever seen consisted of inlays ground from rods of porcelain that you could depend upon for color. Dr. Le Cron selects the color from these rods, or teeth, if you please, and crushes them in the mortar, using the powder from these specimens the color of which he knows. In that way the best results as to color can be reached. It had never occurred to me that this would be practicable, but I shall hereafter make an attempt to match tooth colors by that method.—Dr. C. E. Esterly, in *Western Journal*.

THE CLOSING OF FLASKS.—Never use a large press or a swaging press for closing a flask after packing, for this reason: The plaster of parts when dry absorbs a great deal of moisture when the flask is placed in boiling water for the purpose of softening the rubber, and the rubber not only offers considerable resistance to the saturated plaster, but also condenses it, hence if too much pressure is used the fit is lost; the teeth are likewise easily raised by the thicker rubber at the sides than in other parts before the flask is properly closed, and consequently the bite is thrown out. Always use a small press and do not hurry the closing of the flask.—*Quarterly Circular*.

VERDICT IN SUIT OF INTERNATIONAL TOOTH CROWN COMPANY AGAINST THE HANKS DENTAL ASSOCIATION.—This suit was defended by the Protective Association, and the verdict was in favor of the Crown Co. The issue was the Low bridge patent, which the jury concluded from the evidence, had been infringed, and assessed damages accordingly. The validity of the patent was not finally settled as it was not considered in this case. The Low patent covers the attachment of a dummy tooth to an open face band, or two bands, and the dummy tooth does not touch the gum. It is a question still to be decided whether a saddle bridge or dummies attached to full crowns can be classed as Low bridges.

A VULCANITE BRIDGE.—Dr. Morrison, when he first began to make bridges a great many years ago, down at Sweet Springs, Mo., said that the only satisfactory bridge that could be made would be made by having the abutments of gold and the teeth of rubber. He had some very nice examples of it. I took up the idea and thought it was a great thing. He spoke about rubber being so much more agreeable to use in the masticating surface; that you could get such a perfect masticating surface by taking the bite in wax and making a cast, between the abutments of vulcanizing the bridge, not putting in any teeth at all. After returning home I made a molar bridge, but instead of using the rubber for the masticating surface I used the ordinary rubber teeth and made a saddle of gold, two gold walls attached to the molar and first bicuspid, vulcanizing those teeth into that bridge. That bridge is good to-day. The inner portion, of course, is rubber, and it is cleanly. I see it occasionally, and it is as good a bridge as I can make to-day out of all gold. —Dr. Schulze, in *Western Journal*.

